# Cross-modal associations and synesthesia: Categorical perception and structure in vowel–color mappings in a large online sample

**DOI:** 10.3758/s13428-019-01203-7

**Published:** 2019-04-03

**Authors:** Christine Cuskley, Mark Dingemanse, Simon Kirby, Tessa M. van Leeuwen

**Affiliations:** 1 Centre for Language Evolution, University of Edinburgh, Edinburgh, UK; 2 Max Planck Institute for Psycholinguistics, Nijmegen, The Netherlands; 3 Centre for Language Studies, Radboud University, Nijmegen, The Netherlands; 4 Donders Institute for Brain, Cognition and Behaviour, Radboud University, Nijmegen, The Netherlands

**Keywords:** Synesthesia, Cross-modal correspondences, Language, Sound symbolism

## Abstract

We report associations between vowel sounds, graphemes, and colors collected online from over 1,000 Dutch speakers. We also provide open materials, including a Python implementation of the structure measure and code for a single-page web application to run simple cross-modal tasks. We also provide a full dataset of color–vowel associations from 1,164 participants, including over 200 synesthetes identified using consistency measures. Our analysis reveals salient patterns in the cross-modal associations and introduces a novel measure of isomorphism in cross-modal mappings. We found that, while the acoustic features of vowels significantly predict certain mappings (replicating prior work), both vowel phoneme category and grapheme category are even better predictors of color choice. Phoneme category is the best predictor of color choice overall, pointing to the importance of phonological representations in addition to acoustic cues. Generally, high/front vowels are lighter, more green, and more yellow than low/back vowels. Synesthetes respond more strongly on some dimensions, choosing lighter and more yellow colors for high and mid front vowels than do nonsynesthetes. We also present a novel measure of cross-modal mappings adapted from ecology, which uses a simulated distribution of mappings to measure the extent to which participants’ actual mappings are structured isomorphically across modalities. Synesthetes have mappings that tend to be more structured than nonsynesthetes’, and more consistent color choices across trials correlate with higher structure scores. Nevertheless, the large majority (~ 70%) of participants produce structured mappings, indicating that the capacity to make isomorphically structured mappings across distinct modalities is shared to a large extent, even if the exact nature of the mappings varies across individuals. Overall, this novel structure measure suggests a distribution of structured cross-modal association in the population, with synesthetes at one extreme and participants with unstructured associations at the other.

Cross-modal associations have been proposed as a key mechanism underlying sound-symbolic phenomena relevant to language learning and development (Dingemanse, Blasi, Lupyan, Christiansen, & Monaghan, 2015; Imai & Kita, 2014) as well as to language evolution (Cuskley & Kirby, 2013). Yet, the relationship between cross-modal associations and the much rarer phenomenon of *synesthesia* is still an open area of study. Understanding the relationship between synesthesia and cross-modal associations is a key step in understanding the mechanisms underlying both phenomena, and in particular, their strong relationships to learned categories and language (Cuskley & Kirby, 2013; Simner, 2007). Synesthesia is a relatively rare phenomenon, occurring in approximately 5% of the population (Simner et al., 2006), whereas cross-modal associations are much more widespread. Therefore, capturing the relationship between these phenomena requires examining large samples of participants. This article reports a large-scale study examining the associations between vowel sounds, graphemes, and colors, collected online from over 1,000 participants, simultaneously revealing salient patterns in cross-modal associations and identifying a couple hundred synesthetes in the sample. We begin with an overview of previous studies that have contrasted the associations of synesthetes and nonsynesthetes, with particular relevance to linguistic cross-modal phenomena.

## Synesthesia and cross-modality

Synesthesia, from the Greek *syn*- (together) and -*aisthes* (feeling), is a phenomenon wherein a stimulus in one sensory modality (known as the *inducer*; e.g., a sound) elicits a response not only from that modality (e.g., hearing the sound) but also in another (e.g., *seeing* the sound, known as the *concurrent*). Synesthesia is an involuntary, automatic sensory experience and can occur in many forms, ranging from shaped tastes to smelled colors. Although about a hundred distinct forms of synesthesia have been documented, over 88% of attested forms are linguistic in nature (Simner et al., 2006), with the most widely reported and well-studied form being colored graphemes. In recent years, the study of forms of synesthesia with linguistic inducers has shed light on language processing more generally, showing distinct semantic and phonological effects (Asano & Yokosawa, 2011, 2012; Simner, 2007).

Unlike synesthesia, cross-modal associations are not involuntary or automatic, but nonetheless demonstrate strong relationships between sensory modalities. For example, people reliably associate brighter colors with higher pitch and darker colors with lower pitch (Martino & Marks, 2001). Cross- modal associations are often elicited explicitly (e.g., by asking participants to match a sound to a taste; Simner, Cuskley, & Kirby, 2010), though they have also been demonstrated in implicit contexts (e.g., Ward, Huckstep, & Tsakanikos, 2006). Cross-modal associations are widespread and often shared across a population, though some may vary cross-culturally (Dolscheid, Shayan, Majid, & Casasanto, 2013; Styles & Gawne, 2017).

Despite the commonalities between synesthesia and cross-modal associations, relatively few studies have directly contrasted the two phenomena. In large part, these studies have shown that cross-modal associations in nonsynesthetes show commonalities with inducer–concurrent patterns in synesthetes. Here we focus on a few studies especially relevant to linguistic forms of synesthesia.

An important early study tackling the similarities between synesthesia and cross-modal associations was Simner et al. (2005), which tested associations between colors and graphemes (A–Z as well as 0–9) among English- and German-speaking synesthetes and nonsynesthete controls. Using both forced choice (choosing colors from an array) and open answer (name the color that best matches the grapheme) methodologies, they showed that synesthetes and nonsynesthetes shared many trends in their choices. For instance, both overwhelmingly identified the grapheme *A* as red (cf. Root et al., 2018; Rouw, Case, Gosavi, & Ramachandran, 2014, for recent cross-linguistic confirmations). Using temporally spaced testing (one to three weeks for controls, and two to four months for synesthetes), they also showed that synesthetic participants were far more temporally consistent in their color choices (92%) than controls (32%), despite the longer time interval for testing synesthetes. This study marked the first large-scale demonstration that synesthetes and nonsynesthetes share trends in cross-modal mappings, and it reinforced the idea that temporal consistency is an important feature of synesthesia.

Although graphemes may be the best-known linguistic inducer of synesthesia, it has long been known that phonemes can also play a role, though this remains underexplored (Simner, 2007). In a study of cross-modal associations between vowel sounds and colors, Moos, Smith, Miller, and Simmons (2014) showed that the acoustic properties of F1 and F2 in vowels were significant predictors of color choices in both synesthetes and nonsynesthetes, although more extremely in the former group. F1 and F2 are vowel formants independent of voice pitch (although they are also measured in Hertz), which vary as a result of changing how the vocal tract filters the source sound provided by the vibrating vocal folds. Although not entirely deterministic, changes in F1 and F2 vary predominantly with tongue position in vowel production. The value of F1 is lower when the tongue is higher in the mouth (i.e., high vowels; e.g., the /i/ in *beet*), and higher when the tongue is lower in the mouth (i.e., low vowels; e.g., the /a/ in *bot*). The value of F2, on the other hand, is lower when the tongue is farther back in the mouth, and higher when the tongue is farther forward.^1^ Moos et al. used 16 synthesized vowels that spanned the F1–F2 space and tested 11 English-speaking synesthetes and 20 English-speaking controls. Over hundreds of trials, participants responded to each vowel sound multiple times by choosing one of 16 color swatches, which varied in the dimensions of lightness, green–red, or yellow–blue. They found generally that lower values of F1 (i.e., higher vowels) were greener and yellower, but lower values of F2 (i.e., more back vowels) were more red and blue, and that these trends were much stronger for synesthetes than for controls. Moos et al. concluded that acoustic factors play a privileged role in vowel–color associations over graphemic factors. Similar acoustic-led trends in color choices have been found among nonsynesthetic English–Korean bilinguals (Kim, Nam, & Kim, 2018).

The goal of the present study was to examine the relationship between cross-modal associations and synesthesia in the domain of vowel–color associations among a large online sample of Dutch speakers. Using the same auditory stimuli as Moos et al. (2014), we make several novel contributions to our understanding of cross-modal associations and synesthesia. We aimed to (i) replicate prior results in a larger sample using more fine-grained color measures, (ii) test the novel question of whether categorical perception plays a role in shaping vowel–color associations, and (iii) contribute a new measure of structural isomorphism in cross-modal associations.

In the first instance, we aimed to replicate and extend the results reported in Moos et al. (2014). We used a larger sample (*n* = 1,164) of Dutch speakers and a more fine-grained color response space. Within our sample, we used consistency measures adapted from Rothen, Seth, Witzel, and Ward (2013) to identify synesthetes. Large-scale, online approaches are essential for the study of cross-modality and synesthesia, for several reasons. The large sample allowed us to identify synesthetes behaviorally and to sample more randomly from the population (as opposed to targeted recruitment of synesthetic volunteers, as in, e.g., Baron-Cohen, Burt, Smith-Laittan, Harrison, & Bolton, 1996). This, in turn, provided us with a larger cohort of synesthetes and nonsynesthetes to compare, a distinct advantage for understanding how synesthesia and cross-modality function across populations.

Large sample sizes have been particularly important in earlier studies that have upended our understanding of both the overall prevalence of synesthesia and the skewed sex ratios in the phenomenon. Studies such as Simner et al. (2006) showed that most earlier estimates of the prevalence of synesthesia were off by several orders of magnitude. Early volunteer-recruited studies with small samples had such high F:M ratios that some have suggested that synesthesia might even be X-linked (Baron-Cohen et al., 1996; Smilek et al., 2002). However, recent work with larger, random samples has shown this to be largely an artifact of the volunteer recruitment strategies in earlier studies (Simner & Carmichael, 2015). In short, since the *variation* across the population is a key issue in the study of cross-modality and synesthesia, a large-scale online approach is essential for a more complete understanding of the phenomena.

The large-scale online approach also presents some challenges. Tasks generally need to be shorter in order to maximize the number of participants completing the task, and the circumstances of participation are less controlled than in a lab setting. Unlike in Moos et al. (2014), the task reported here consisted of only three trials per vowel item, and we expected that our data might be noisier as a result of less controlled participation conditions. To the extent that the methods introduced noise, this should stack the deck against our hypotheses of finding acoustic, categorical, and cross-modal structure in vowel–color associations, rendering any findings of structure more robust. Also, as in other online experiments in behavioral science, we expected increased noise in the data to be offset by the considerable advantages of having such a large sample (Crump, McDonnell, & Gureckis, 2013).

A second aim of our study was to look beyond acoustic or graphemic factors and examine how vowel phoneme category might influence associations in both synesthetes and nonsynesthetes. While we expected to replicate Moos et al.’s (2014) results regarding acoustic factors, a key focus of our study was the role of *categorical perception*. We hypothesized that since vowel perception is predominantly categorical (Rosner & Pickering, 1994), vowel category may be a better predictor of color choices than are acoustic factors. We predicted that acoustic factors would be a good predictor of color choices insofar as changes in acoustic structure correlated with changes in vowel category. Given the prevalence of grapheme–color synesthesia, the best-approximated vowel grapheme might also form a strong predictor, especially for participants who are grapheme–color synesthetes. However, grapheme category might also play an influential role for nonsynesthetes, since auditory information can automatically invoke graphemic form (Cuskley, Simner, & Kirby, 2017; Ziegler & Ferrand, 1998).

Finally, we presented a novel measure of isomorphic structure in cross-modal associations using the Mantel test (Mantel, 1967). We argue that this measure forms a useful complement to existing consistency tests used to identify synesthetes, especially in large samples. This measure quantifies the extent to which an individual’s associations are isomorphically structured (i.e., similar sounds are matched with similar colors) or unstructured (i.e., similar sounds are matched with dissimilar colors). We used this measure to show that while the specific nature of cross-modal mappings may exhibit considerable individual variation, the *capacity* for making structured mappings is shared to a much broader extent in the population. This new measure of structure in cross-modal associations provides a promising way to probe structural isomorphism across domains or sensory modalities generally.

## Method

### Participants

A total of 1,164 adult participants volunteered to take part in an online vowel–color association task as part of a larger survey on cross-modal associations advertised in the Dutch national press, popular media, and Dutch national TV as the “Groot Nationaal Onderzoek” (“Large National Survey”; van Leeuwen & Dingemanse, 2016). This is an annual public engagement initiative of the national broadcaster NTR, aiming to actively involve the general public in research. The funding attached to this initiative enabled us to design and develop a dedicated web application, and widespread advertisement in the national media facilitated the large sample size.

Participants provided informed consent prior to participation and were not required to provide any personal information. About 85% of participants provided some information in a voluntary pretask survey that could be answered anonymously. The reported age range was 18–88 years old (median = 46, *SD* = 16); reported gender was 667 female, 206 male, and 282 who did not select a gender.^2^ A subset of 398 participants additionally carried out a grapheme–color association task.

### Materials

The stimuli in the vowel–color association task were 16 vowel sounds selected to represent points spread through acoustic vowel space. Moos et al. (2014) reported the method for creating these stimuli as follows:

Recordings of the eight primary cardinal vowels were made in a high-specification sound studio. . . . To create a richer vowel continuum, eight intermediate vowels were made by morphing each neighbouring pair. . . . The 16 vowels were adjusted in intensity (to 80 dBSPEL) and duration (to 1049 ms, the mean duration of the original stimuli) using Praat’s PSOLA function Boersma and Weenink (2011). F0 [which corresponds to voice pitch] varied minimally, from 120 to 124 Hertz, and was not equalised. (Moos et al., 2014, p. 134)

The stimuli in the grapheme–color association task were the characters A–Z (capital) and 0–9 presented in black sans-serif capitals on a gray background. In each task, items were presented in randomized order and each item occurred three times, making for a total of 48 trials in the vowel task and 108 trials in the grapheme task. Consistency scores across trials per item and task were calculated in order to identify synesthetes in the sample (described in further detail below).

Color responses were recorded using an RGB color picker following widely used methods in online test batteries of synesthesia (Eagleman, Kagan, Nelson, Sagaram, & Sarma, 2007; Rothen et al., 2013). Even though RGB values are device-specific, data collected using RGB color pickers are robust enough for the detection of synesthesia, especially when converted to the more perception-veridical CIE color space (Rothen et al., 2013) and analyzed in terms of relative distances. For the model analyses below, we converted the RGB color responses to CIELuv space (as in Moos et al., 2014) using the standard illuminant D65, particularly in order to use the CIELuv-based consistency measures from Rothen et al.’s study.

### Procedure

Both association tests were available online. To maximize participation while assuring high-quality data, we piloted the tasks across multiple platforms, making sure they would run smoothly across devices without loss of functionality or data. The instructions were kept as clear and concise as possible and were similar to those provided for similar tasks in the lab and on other synesthesia-screening websites. Full code for the web application used to collect the data is available at http://github.com/mdingemanse/colouredvowels.

For the vowel–color task, the instructions were as follows: “In this test you associate colors to sounds. You hear a sound, then choose a color that you feel fits best. Synesthetes always see the same color for the same sound: is that the same for you? Try to respond as intuitively as possible.” For the grapheme–color task, the instructions were as follows: “In this test you associate colors to letters in a precise way. You see a letter or digit, then choose a color that you feel fits best. Synesthetes always see the same color for a letter or digit. Does this hold for you, too? Try to respond as intuitively as possible.”^3^

**Fig. 1:**
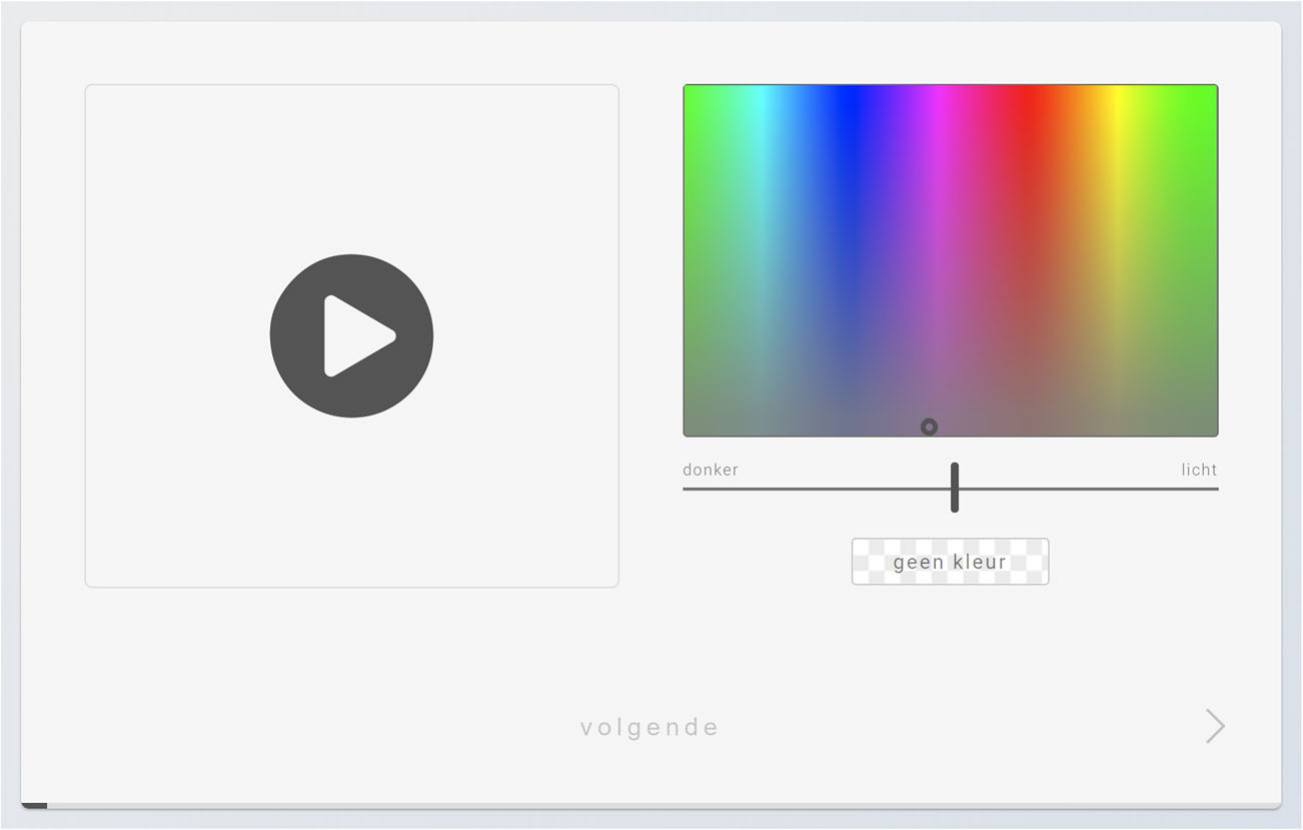
Screenshot of the interface for the vowel–color association task

In the vowel–color task, participants first were presented with a brief sound test to check their audio volume. Next, the 16 vowel items were presented in a random order three times, for a total of 48 trials. Each trial began with the audio file autoplaying. After the file had played once, participants could relisten to each audio file as many times as they wanted before choosing a color. In the grapheme–color task, the 36 items (the letters A–Z and digits 0–9) were presented in a random order three times, for a total of 108 trials. In both tasks, participants clicked on the “Next” button after choosing their desired color in order to continue to the next trial. The interface for the experiment during a vowel trial is shown in Fig. 1.

In both tasks, the RGB spectrum of the color picker was randomly shifted on the horizontal axis after each trial, making it difficult to achieve high consistency just by clicking in the same region of the color picker (i.e., making a spatial association). A separate bar allowed participants to adjust the lightness of the chosen color. On each trial, it was possible to choose “no color.” This option was used in less than 5% of trials in the vowel color task, and in around 11% of trials in the grapheme–color task.

### Data preparation

The test data were logged locally in the participant’s browser and transmitted to an SQL database on a secure server upon task completion. The results were postprocessed offline in R. To safeguard against possible duplicate data entries from the same individuals, responses from the same email address or same name, or from the same IP address without differential name or email address information, were removed. Overall, 11 completed tests were detected as duplicates and removed. Immediately after these quality control steps, the data were anonymized, keeping a random identifier as the sole link between the anonymized demographic metadata and test results.

The color responses in both tasks were converted to CIELuv values as in Moos et al. (2014), using Python’s colormath package (Taylor, 2017). CIE spaces are generally preferable to RGB space because they are based on how people perceive color (rather than, e.g., how a computer should render it). CIELuv distances in particular have been shown to be more accurate in detecting synesthetes (Rothen et al., 2013) than RGB distances (Eagleman et al., 2007). In CIELuv space, *L* corresponds to lightness, *u* corresponds to a green–red continuum, and *v* corresponds to a blue–yellow continuum.

Following Rothen et al. (2013), we calculate a consistency score for each participant as follows. First, we calculated *d* (Eq. 1) as the sum of the paired Euclidean distances in CIELuv space between the three color choice trials for each vowel or grapheme (see the equation below, adapted from Eq. 2 in Rothen et al., 2013, p. 158). The *d* values for each item were used to create a mean *c* across items for each participant, which served as their CIELuv consistency score. Following Rothen et al., we defined synesthetes as those with consistency *c* < 135.3.

1 $$ d={\sum}_{\left(i=1,2,3\right)}\sqrt{{\left({L}_1-{L}_2\right)}^2+{\left({u}_1-{u}_2\right)}^2+{\left({v}_1-{v}_2\right)}^2}, $$

**Fig. 2:**
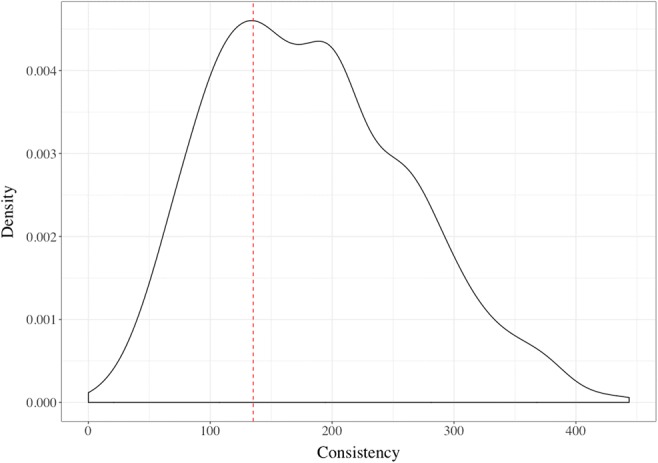
Density plot of all consistency scores for vowel–color associations. The red dashed line indicates the cutoff for synesthesia from Rothen et al. (2013), with all participants to the left of the line being classified as vowel–color synesthetes

A total of 34 participants were removed from the sample because they chose “No color” for more than half of the items in the vowel association task, making it impossible to calculate a valid vowel consistency score. In the remaining participants, we identified 365 vowel–color synesthetes, with a mean *c* = 95.60 (*SD* = 28.00, 95% CI = 2.88). The nonsynesthetes had a mean of *c* = 224.33 (*SD* = 63.47, 95% CI = 4.5). Figure 2 shows the overall distribution of consistency scores, with the dashed red line indicating the consistency cutoff used in Rothen et al. (2013).

Since we lacked grapheme consistency data for much of the sample, the participants who completed the grapheme association task but did not provide data sufficient for consistency calculations were retained in the sample and classified as unknown for grapheme color synesthesia, along with participants who did not complete the grapheme task. The grapheme synesthetes had a mean *c* = 82.45 (*SD* = 38.91, 95% CI = 9.21) on the grapheme task, a value only slightly lower (higher consistency) than that reported by Rothen et al. (2013, p. 160; *c* = 85.51, *SD* = 58.27, CI not reported). Nonsynesthetes had a mean *c* = 243.19 (*SD* = 64.83, 95% CI = 10.03), a value slightly higher than the one reported by Rothen et al. (p. 160; *c* = 219.38, *SD* = 68.87, CI not reported).

Table 1 shows the synesthetic status of the 1,130 participants based on the *c* cutoff value in each task. The rate of synesthesia in our sample, for both grapheme–color and vowel–color synesthesia, is significantly higher than would be expected in a random sample (e.g., Simner & Carmichael, 2015; Simner et al., 2006). This is likely because the task was specifically advertised for people interested in cross-sensory associations and synesthesia, resulting in higher participation rates of synesthetes than in a truly random sample (Dingemanse & van Leeuwen 2015).

**Table 1:** Synesthetes and nonsynesthetes in the sample, as defined by the consistency score cutoffs reported in Rothen et al. (2013)

		Vowel–Color		Total
		Nonsynesthete	Synesthete	
Grapheme–Color	Synesthete	38	33	71
	Nonsynesthete	114	49	163
	Unknown	613	283	896
	Total	765	365	

**Fig. 3:**
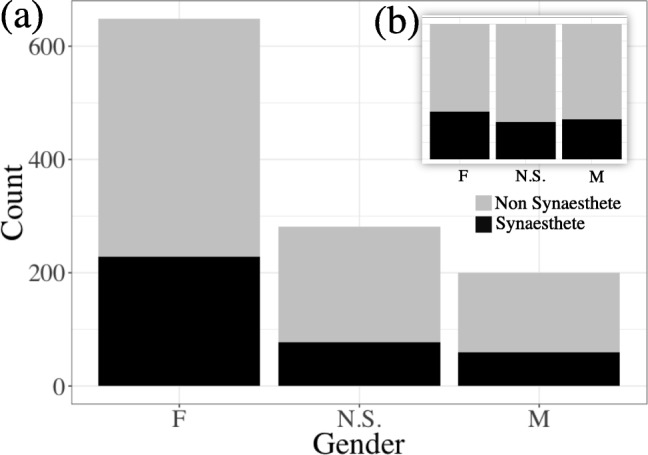
Rates of synesthesia, according to consistency scores, among female (F), male (M), and other (N.S., not selected) participants in our sample. The inset shows the proportions for each group, showing that despite the greater number of females overall, the rates of synesthesia among the three groups were comparable

Although the overall prevalence of synesthesia in our sample was likely skewed, we did not find a significantly higher proportion of female synesthetes, though we did have more female participants overall (Fig. 3). This echoes recent studies showing that the early reports of much higher rates of synesthesia in females were likely due to sampling methods that relied on targeted recruitment of self-reported synesthetes (Simner & Carmichael, 2015), as contrasted with the present study, which invited participants interested in cross-modality associations generally. The use of consistency scores to automatically identify synesthetes without self-report may also have resulted in false positives, an issue we will address in part with our structure measure.

To examine the role that categorical perception might play in respondents’ color choices relative to acoustic measures, we used the F1 and F2 values of the vowel stimuli provided by Moos et al. (2014) as our acoustic predictor. For categorical phoneme perception, each of the stimuli was categorized as a Dutch vowel phoneme. Using F1–F2 values from Dutch vowels taken from Adank, van Hout, and Smits (2004), each stimulus was categorized as an instance of its nearest Euclidean neighbor in F1–F2 space, on the basis of the overall mean F1–F2 values for each monophthong Dutch vowel.

**Fig. 4:**
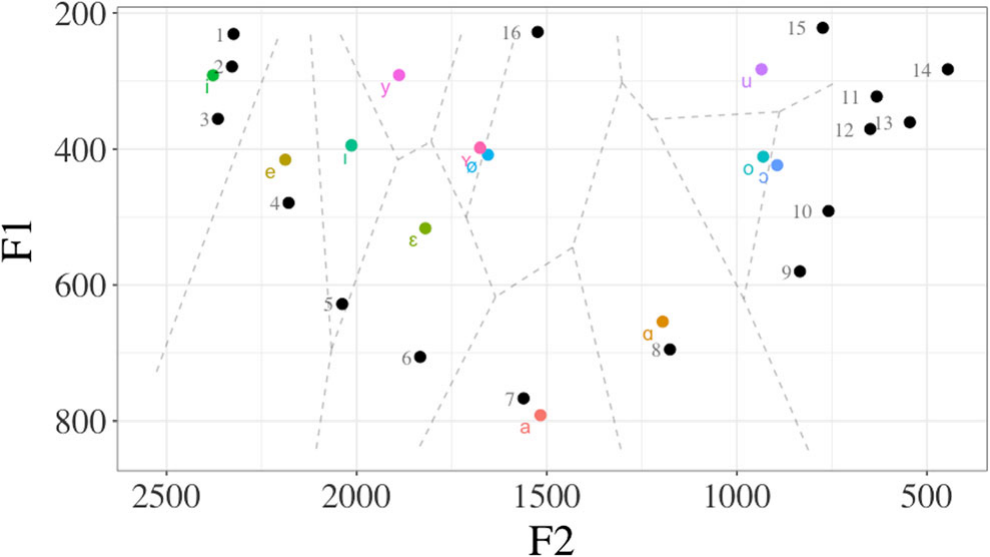
Canonical Dutch monophthong vowels plotted in F1–F2 space, taken from Adank et al., 2004. The dashed lines indicate a Voronoi tesselation: Each cell contains all points that are closest to the canonical vowel within that cell. Thus, the vowel stimuli (numbered and indicated by black dots) were classified according to their nearest phoneme neighbor in Euclidean space

Figure 4 shows each Dutch vowel phoneme plotted in F1–F2 space. We used a Voronoi tesselation (dashed lines), such that every point within each cell is closer to the central phoneme of the cell than to any other phoneme (measured in Euclidean distance). Vowel stimuli were categorized according to the cell they fell into, detailed in Table 6 in Appendix 1, which also details their F1–F2 values and grapheme category (according to the general Dutch orthography conventions from Nunn, 2006, p. 15).

## Results

### Model analyses

We used linear models to analyze participants’ responses in each dimension of the CIELuv space, adopting a mixed-effect approach to account for repeated measures, with random effects for participants and trial. Where individual fixed effects or interactions did not significantly improve the model fit, they were dropped, using the step() function from the lmerTest package (Kuznetsova, Brockho, & Christensen, 2018). All of the models reported below have significantly improved fit over a null model or simpler alternatives. The models were analyzed in R using the lme4 package (Bates, Maechler, Bolker, & Walker, 2013); *F* and *p* values were estimated using Straitherwaite approximations and the anova() function in the lmerTest package (Kuznetsova et al., 2018).

We examined the continuous predictors of F1 and F2 vowel formants, as well as the categorical predictors of vowel phoneme category and grapheme category, summarized in Table 2. We compared models with different predictors using the sem.model.fits function in the piecewiseSEM package (Lefcheck, 2016), which compares Akaike information criterion (AIC) values across models and estimates the marginal (fixed effect) and conditional (fixed + random effect) *R*^2^ values for each model. Although phonemic, graphemic, and acoustic factors all play roles, we found that vowel phoneme category is the best overall predictor of color choice, and that grapheme category accounts for more variation than acoustic factors. Details of the grapheme models are included in Appendix 2, but they will not be discussed at length here, since vowel category is a better predictor of responses. In the following analyses, we focus first on acoustic factors for a comparison with earlier work, followed by further analyses of vowel phoneme category.

**Table 2:** Marginal and conditional *R*^2^ values for each predictor in each dimension of color space

		Marginal *R*^2^	Conditional *R*^2^	dAIC	Fixed effects
*L*	Acoustic	.155	.336	456	F1 + F2 + vSyn + F2 × vSyn + F1 × vSyn
	Vowel	.161	.342	–	vowel × vSyn
	Grapheme	.154	.340	463	grapheme × graphemeSyn
*u*	Acoustic	.017	.131	379	F1 + F2 + vSyn + F2 × vSyn + F1 × vSyn
	Vowel	.024	.138	–	vowel × vSyn
	Grapheme	.023	.136	62.12	grapheme × graphemeSyn
*v*	Acoustic	.080	.219	264	F1 + F2 + vSyn + F2 × vSyn
	Vowel	.085	.223	–	vowel × vSyn
	Grapheme	.081	.220	190	grapheme × graphemeSyn

dAIC stands for delta AIC (Akaike information criterion), which is an estimate of fit. Lower values of AIC indicate better model fit; where this number is omitted, it indicates the lowest AIC value. For all models, trial and participant were included as random effects. In the fixed effects described in the last column, *vSyn* stands for vowel synesthete, and *graphemeSyn* stands for grapheme synesthete, whereas *vowel* and *grapheme* indicate the respective categorical predictors. In all dimensions, vowel category predicts the most variation in responses.

#### Acoustic factors

**Fig. 5:**
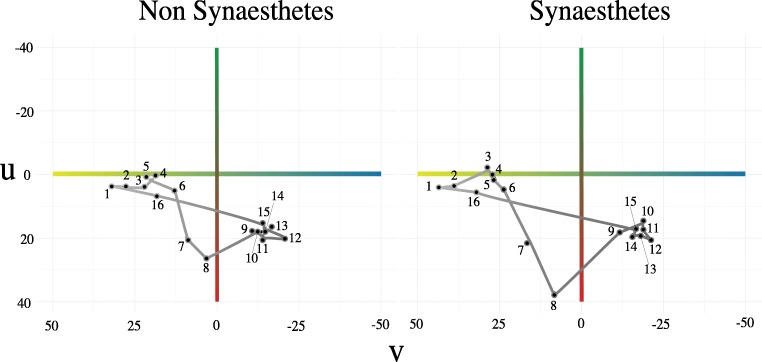
Vowels plotted in color space for nonsynesthetes (left) and synesthetes (right), after Moos et al. (2014). Note that the colors represented here are slightly idealized, for comparison with the plots in Moos et al. (2014), and the lightness choices indicated are an approximate gradient interpolation between the lightest and darkest values chosen (i.e., the midrange values are not exact). For more precise color and lightness choices, see Fig. 6

**Fig. 6:**
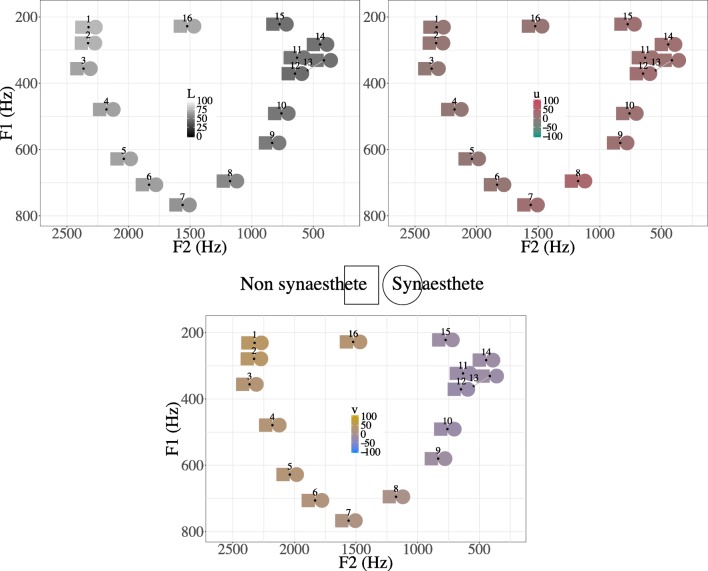
Mean *L* (top left), *u* (top right), and *v* (bottom) color values chosen for vowel items, as plotted in F1–F2 space for synesthetes (squares) and nonsynesthetes (circles). Axis labels indicate vowel quality (vowel height on the F1 axis and front/back vowels on the F2 axis). For each color scale, the values shown reflect the mean for the item in the relevant dimension, with the other dimensions held at middle values (the middle values for *L*, *u*, and *v*, are 50, 0, and 0, respectively)

Figure 5 shows the results in color space in the same way as in Moos et al. (2014). As in Moos et al.’s study, we found that synesthetes chose a generally wider range of colors, but the general shape of the mapping between vowels and colors was shared between synesthetes and nonsynesthetes. Figure 6 shows more exact color values chosen in each dimension for each item, plotted in F1–F2 space.

In the formant models described below, F1, F2, and synesthetic status were tested as fixed-effect predictors of *L*, *u*, and *v*. The models tested interactions with each acoustic predictor and synesthetic status (but not between acoustic predictors) as fixed effects, after Moos et al. (2014). Below we describe the results for *L*, *u*, and *v*, respectively.

For acoustic factors, more variance was accounted for in lightness choices (~ 15%, marginal *R*^2^ = .155) than in the *u* and *v* dimensions, where the fixed effects accounted for less than 10% of variation (*u*, red–green, marginal *R*^2^ = .017; *v*, blue–yellow, marginal *R*^2^ = .08).

Participants generally chose lighter colors for more front vowels and darker colors for more back vowels (Fig. 6, top left), reflected by a strong significant main effect of F2 in the model (Table 3). The model also showed that participants chose slightly lighter colors for lower vowels. Synesthetes were likely to choose slightly darker colors overall, but an interaction between F2 and synesthetic status showed that synesthetes tended to choose slightly lighter colors for front vowels than nonsynesthetes. On the other hand, the interaction between F1 and synesthetic status showed that synesthetes chose slightly darker colors for lower vowels (higher values of F1) than nonsynesthetes. Although the synesthete/nonsynesthete contrasts are strongly significant, they are difficult to detect in Fig. 6, since the color swatches reflect means of choices from a large, continuous color palette instead from a predefined set of 16 colors, as in Moos et al. (2014). For the specific estimates and *p* values of the fixed effects, see Table 3. For examples of the individual response patterns, see Fig. 10 below.

**Table 3:** Model: *L* ~ F1 + F2 + vSyn + F2 × vSyn + F1 × vSyn + (1 | Participant) + (1 | Trial)

	Est.	*SE*	*F*	*p*
(Intercept)	43.62	0.454	–	–
F1	0.001	0.0004	5.55	.018
F2	0.010	0.0001	11,318	< .001
vowelSyn	– 1.80	0.728	6.11	.013
F1 × vowelSyn	– 0.003	0.001	248	< .001
F2 × vowelSyn	0.004	0.0002	24.56	< .001

In terms of the *u* (green–red) scale (Fig. 6, top right), acoustic factors accounted for the least amount of variation in the *u* (green–red) scale, with less than 2% of the variation in responses being predicted by variation in F1, F2, and synesthetic status. Participants generally chose redder colors for lower vowels (higher F1) and more back vowels (higher F2). There was no main effect of synesthetic status in this dimension; however, there were significant interactions with F1 and F2. Synesthetes chose colors that were greener for low and back vowels than did nonsynesthetes. The detailed estimates, along with *F* and *p* values, are outlined in Table 4.

**Table 4:** Model: *u* ~ F1 + F2 + vSyn + F2 × vSyn + F1 × vSyn + (1 | Participant) + (1 | Trial)

	Est.	*SE*	*F*	*p*
(Intercept)	20.16	1.09	–	–
F1	0.012	0.001	126	< .001
F2	– 0.009	0.0004	864	< .001
vowelSyn	– 0.2295	1.93	0.01	.905
F1 × vowelSyn	0.006	0.003	5.37	.020
F2 × vowelSyn	0.001	0.0007	4.43	.035

On the blue–yellow (*v*) dimension (Fig. 6, bottom), the interaction between F1 and synesthetic status was dropped, since it did not improve model fit. Participants preferred yellower colors for high (low F1) and front (high F2) vowels. Synesthetes preferred bluer colors overall; however, they chose significantly yellower colors for front vowels than did nonsynesthetic participants, reflected in the significant interaction between F2 and synesthetic status. The detailed estimates, along with *F* and *p* values, are outlined in Table 5.

**Table 5:** Model: *v* ~ F1 + F2 + vowelSyn + F2 × vowelSyn + (1 | Participant) + (1 | Trial)

	Est.	*SE*	*F*	*p*
(Intercept)	– 26.72	1.26	–	–
F1	0.005	0.001	12.17	< .001
F2	0.024	0.0004	860.09	< .001
vowelSyn	– 6.25	1.99	2.201	.002
F2 × vowelSyn	0.007	0.0007	3.35	< .001

#### Phonemes and graphemes

In the categorical predictor models described in this section, phoneme (and grapheme) category and synesthetic status were fixed-effect predictors of *L*, *u*, and *v*. As with acoustic predictors, these models also tested interactions between phoneme (and grapheme) category and synesthetic status as fixed effects. For grapheme category, grapheme synesthete status was used as the synesthetic predictor instead of vowel synesthete status. Since vowel category was a better predictor of responses, we will not detail the grapheme results here, but they are provided in Appendix 2. The detailed results of the vowel models are provided below.

For vowel category, we observed significant main effects of this variable in all three color dimensions (*L*, *F* = 1,493, *p* < .001; *u*, *F* = 166, *p* < .001; *v*, *F* = 660, *p* < .001), as well as a significant main effect of synesthetic status in the *L* and *v* dimensions (*L*, *F* = 10.73, *p* = .001; *u*, *F* = 0.75, *p* = .39; *v*, *F* = 660, *p* < .001). These results echo those found for acoustic factors, with synesthetes choosing lighter (estimate = 1.34, *SE* = 0.49) and yellower (estimate = 6.92, *SE* = 2.71) colors than nonsynesthetes.

**Fig. 7:**
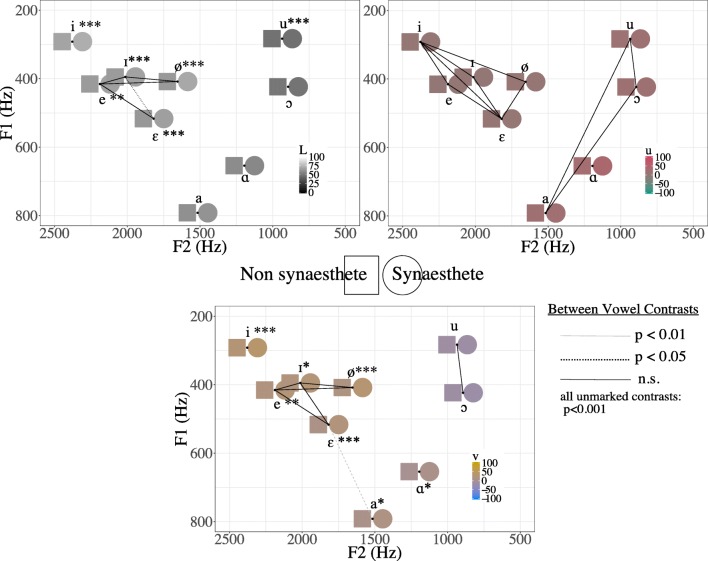
Mean color choices for and contrasts between vowel categories plotted in F1–F2 space, labeled with canonical Dutch phoneme values from Adank et al. (2004). The axis labels indicate vowel quality (vowel height on the F1 axis, and front/back vowels on the F2 axis). For each color scale, the values shown reflect the mean for the item in the relevant dimension with the other dimensions held at middle values (the middle values for *L*, *u*, and *v*, are 50, 0, and 0, respectively). Solid lines between vowels indicate nonsignificant contrasts, as per the legend. Thus, clusters of vowels connected by lines did not elicit significantly different color choices from participants, and can be interpreted as informal groups. Asterisks next to a phoneme indicate that synesthetes and nonsynesthetes chose significantly different colors for that phoneme (see the text for details)

To assess specific effects for vowels, we calculated contrasts between all vowel categories in each dimension, with Bonferroni adjustments for all reported *p* values, using the lsmeans package (Lenth, 2016). The differences between vowel categories were mostly highly significant for all color dimensions and are fully listed in Appendix 3, with estimated means, confidence intervals, *t* ratios, and *p* values. Since the contrasts were mostly significant, Fig. 7 shows which vowels were *not* different from one another, using black lines for uncorrected nonsignificant contrasts (*p* > .05), a dotted line for a contrast in which *p* < .05, and a dashed line for a contrast in which *p* < .01. In other words, the more solid a line between two vowels is, the more similarly participants responded to them. For all unmarked contrasts, *p* < .001. Vowels are plotted with their canonical Dutch phoneme values from Adank et al. (2004). Figure 7 shows the results in each dimension.

In all dimensions, the vowels /e, , ε, ø/ tended not to be significantly different, forming a “mid- front” grouping for which participants generally chose similar colors that were lighter, greener, and yellower than those of low back vowels. In both the *L* and *v* spaces, the vowel /i/ stood apart from this group, being even lighter and yellower. The vowels /u/ and // were also informally grouped in the *u* and *v* dimensions. In the *u* dimension, these also converged with /a/, although /ɑ/ was set apart as the reddest of all the vowels.

Contrasts between synesthetes and nonsynesthetes were not calculated for the *u* dimension, due to the lack of a main effect of synesthetic status. For the other dimensions, significant contrasts between synesthetes and nonsynesthetes are marked in Fig. 7 as follows: ^***^p < .001, ^**^p < .01, ^*^p < .05. The vowels in the “mid-front” grouping described above were generally lighter for synesthetes than for nonsynesthetes, and this was also true for the highest front vowel /i/. In the *v* dimension, synesthetes’ choices were generally yellower than nonsynesthetes’ for the same “mid-front” grouping, and also yellower for the high front vowel /i/. They were also slightly yellower for the low vowels /a/ and /ɑ/.

#### Summary

In a large sample of Dutch speakers, we found evidence of shared vowel–color associations. As in earlier work, our data showed that the acoustic factors F1 and F2 were predictive of color choices: Higher values of F1 (i.e., lower vowels) are darker, redder, and bluer; higher values of F2 (i.e., more front vowels) are lighter, greener, and yellower. These results echo those found for English speakers by Moos et al. (2014), and for Korean–English bilinguals in Kim et al. (2018). Although the general shape of associations is shared across synesthetes and nonsynesthetes (Fig. 5), synesthetes show more extreme color and lightness choices, selecting lighter and yellower colors for high values of F2 especially. The differences between synesthetes and nonsynesthetes in our results are not as marked as those reported by Moos et al.; we address several potential reasons for this in the General Discussion.

However, we also found that an approximation of phoneme category is a better predictor of color choice than the acoustic measures, indicating that categorical perception can shape the structure of cross-modal associations. As would be expected, given the acoustic results, front vowels are lighter, greener, and yellower, whereas low and back vowels are darker, redder, and bluer. There were generally no significant differences between the “mid-front” group of /e/, //, /ø/, and /ε/. However, particularly for this group, synesthetes chose slightly lighter and yellower colors than did nonsynesthetes. They also chose lighter and yellower colors for the high front vowel /i/, and darker colors for the low back vowel /u/.

Although the values from the phoneme model are very close to those from the grapheme model, comparisons of the two predictors showed that the phoneme model is significantly superior in every color dimension. It may be that grapheme category is only a good predictor of color choices insofar as it is a fairly good, though imperfect, predictor of vowel category. Although a rough mid-front vowel grouping emerged in the phoneme category analyses, this does not correspond to a larger grapheme grouping, since the four relevant phonemes map onto three different graphemes (/e/ and /ε/ to *e*, // to *i*, and /ø/ to *u*) in Dutch orthography.

### Mapping structure

So far, our analyses were largely concerned with the kind of questions asked in classic cross-modal association studies, linking specific colors or color dimensions to acoustic and phonemic features. We also looked at contrasts between synesthetes and nonsynesthetes, based largely on consistency across trials. Although the temporal consistency of mappings is rightly considered a benchmark of genuine synesthesia, and some earlier studies have considered how the mappings of synesthetes relate to those of nonsynesthetes, less consideration has been given to the internal structure of synesthetic and cross-modal mappings. The traditional approach tells us something about whether synesthetes choose colors for sounds that are different from or similar to the kinds chosen by nonsynesthetes, but it is less adept at detecting overall structure in cross-modal mappings or telling us whether the mappings of synesthetes are more internally structured than those of nonsynesthetes. Are there structural regularities in how we link one sensory domain to another? Does the shape of the vowel space map onto the color space more reliably for synesthetes than for nonsynesthetes?

We operationalized structure in this context by comparing paired distances across spaces, using a method borrowed from ecology (Mantel, 1967). This method has been used extensively in iterated artificial-language learning studies to detect structured mappings between form and meaning spaces (e.g., Kirby, Cornish, & Smith, 2008). The Mantel test in this context measures whether distances in form correlate with distances in meaning. To the extent that they do, we can say there is structure in the mappings between two spaces.

In the context of the present data, for example, a mapping would be structured when pairs of vowels that are similar in F1–F2 acoustic space map onto pairs of colors that are similar in three-dimensional color space, and when pairs of vowels that are dissimilar in F1–F2 acoustic space map onto pairs of colors that are dissimilar. Thus, structure implies a degree of isomorphism across (multidimensional) sensory spaces—in this case, acoustic and color spaces.

Whereas our earlier consistency measures had used CIELuv space to align with prior work (Rothen et al., 2013), for this measure we used the related CIELab space. In CIELab space, the *L* dimension is identical, whereas *a* corresponds to a green–red continuum (similar to *u*) and *b* corresponds to a blue–yellow continuum (similar to *v*). The benefit of CIElab space for the structure measure is that it allows us to use more perceptually realistic distances, specifically ∆*E*_2000_ (Sharma, Wencheng, & Dalal, 2005). The ∆*E*_2000_ distance takes into account that Euclidean distances have nonuniform perceptual effects, particularly at the edges of the color space. For example, as lightness increases to the white point, the perceptual differences between chroma shrink and eventually disappear, even though their plain Euclidean distance in the CIELab or CIELuv spaces would be identical to those of two perceptually distinct colors elsewhere in the space. Therefore, our structure measure relies on ∆*E*_2000_ distances in CIELab space. For vowel distances, we used Euclidean distance in F1–F2 space using the canonical phoneme values shown in Fig. 7.^4^ Since Euclidean distance can be high-dimensional, this allowed us to use all three dimensions of a participants’ color response to an item at once.

Where there is structure, the pairwise distances within each space will be correlated with one another. Once we had pairwise distances for every mapping in each space from our participants’ data, we permuted the vowel–color mapping between the two spaces and then recalculated the pairwise distances in order to get a distribution of potential correlations between the two spaces. Using this distribution, we obtained a *z* score indicating where a given participant’s mappings were on the actual distribution, and a *p* value that indicated the likelihood that any random mapping of the vowel–color space would be more structured than the actual one.^5^ In other words, *p* < .05 in this case means that fewer than 5% of mappings generated in the simulation were more structured than the real one.

**Fig. 8:**
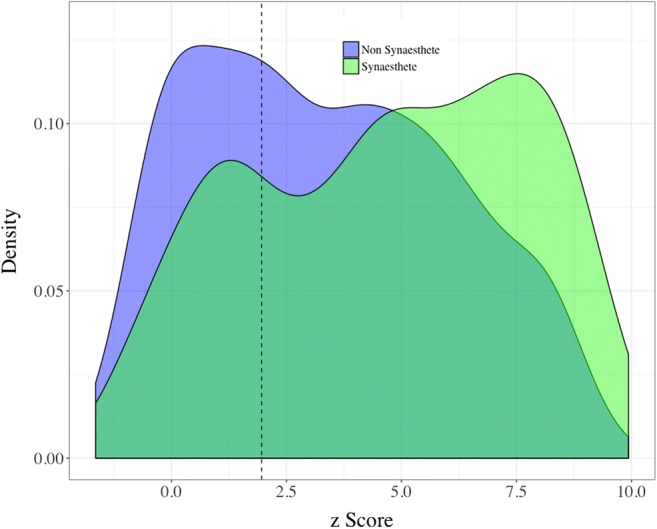
Distributions of structure scores among synesthetes and nonsynesthetes. Higher *z*-score values indicate more structured mappings. Values to the right of the dashed line indicate mappings that are significantly structured

Pairwise distances were calculated between all sounds and all colors chosen by a particular participant, and a veridical correlation was calculated between these distance matrices. To account for multiple responses to the same stimuli, the responses were first shuffled within a particular item, and then across items. These matrices were then shuffled into 10,000 random permutations, each with its own *r*, allowing us to calculate a *z* score as described above. Python code for performing the Mantel structure analyses is available online at http://github.com/mdingemanse/colouredvowels. Figure 8 shows a density plot of *z* scores of synesthetes and nonsynesthetes in the vowel–color association task.

**Fig. 9:**
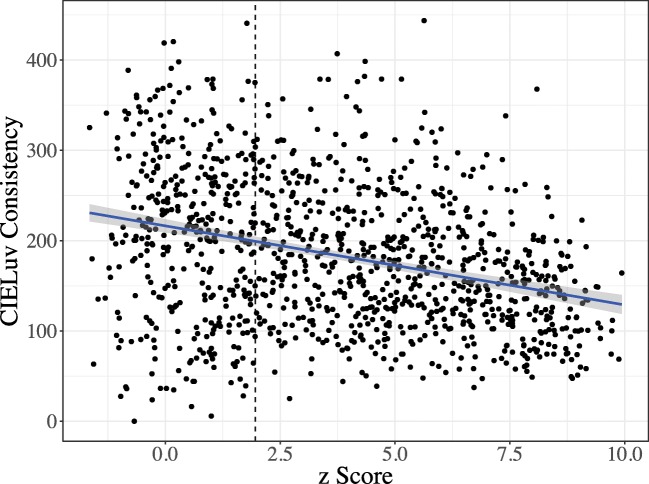
Relationship between structure score and consistency score, showing that participants with more consistency (i.e., lower CIELuv color distance) across trials tend to have more structured mappings (i.e., higher *z* scores)

Three findings stand out. First, the mappings of synesthetes tend to be more structured than those of nonsynesthetes (*t* = – 7.09, *df* = 660, *p* < .001). Second, the majority of participants’ mappings are more structured than would be expected by chance: All participants to the right of the vertical dotted line had correlations between the vowel and color spaces greater than 95% of random permutations generated by the Mantel test. Third, there is correlation between the structure score and CIELuv consistency scores: Participants with more consistent associations across trials (i.e., lower consistency scores) tended to have more structured mappings across the vowel and color spaces (*r* = – .313, *t* = – 11.07, *p* < .001; Fig. 9).

**Fig. 10:**
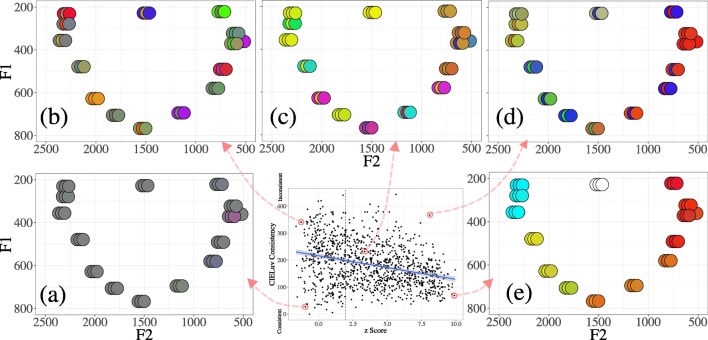
Mappings of individual participants showing, clockwise from bottom left, (a) a participant with very low structure yet high consistency across trials and items, probably indicating a false positive synesthete, (b) a typical nonsynesthete with inconsistent and unstructured mappings, (c) a middling participant with significant structure but inconsistent choices across trials, and (d) a highly structured but inconsistent participant, and (e) a typical vowel–color synesthete, with highly structured, consistent and categorical mappings

This measure provides a new way to quantify the structure of cross-modal mappings and is a valuable quantitative complement to traditional unimodal consistency scores. Figure 10 shows individual participants that fall in specific parts of the consistency-structure space. The participant in panel a, who was classified as a synesthete according to consistency, appears to have achieved this by having high consistency across items (i.e., choosing the same color regardless of stimulus or trial), rather than by having structured, temporally consistent associations. This indicates that participants with high consistency and low structure are less likely to be genuine synesthetes, perhaps explaining the slight peak of unstructured synesthetes in Fig. 8. The participant in panel b has both low structure and low consistency, having chosen idiosyncratic colors on each trial and across the space, and sometimes mapping distant vowels (e.g., low-central and high-back/high-front vowels) with similar colors.

A nonsynesthete participant with middling consistency and significant but not especially high structure is shown in panel c. This participant shows structured mappings for some parts of the space—for instance, showing similar yellow/green choices for the cluster of high-front vowels, and brownish choices for the cluster of high back vowels, resulting in significant structure. However, this participant was inconsistent across trials for the same item, distinguishing the participant from highly structured synesthetes like the participant in panel e, showing categorical, structured associations that are highly consistent across trials. Finally, panel d shows a participant with high structure but low consistency: This participant made structured mappings across the space, but seems to have done so differently on each trial, as indicated by the inversions of green/blue in mid-front vowels and red/blue in back vowels across trials.

## Discussion

We examined vowel–color associations in a large number of participants in an online study. Four key findings emerge.

First, acoustic factors (F1 and F2) predict some of the variance in color responses, replicating a result by Moos et al. (2014), but in a different and larger population. Participants chose lighter colors for more front vowels (i.e., vowels with higher F2 values), redder colors for lower vowels (i.e., vowels with higher F1 values), and greener colors for high and front vowels (i.e., lower F1 and higher F2 values, respectively). Synesthetes showed the same patterns but were slightly more extreme, choosing even lighter and yellower colors for high vowels in particular, an effect also found by Moos et al. Overall, the locations of associations in the color space were not identical to those found by Moos et al., but the general differences in shape were comparable: Synesthetes’ choices were generally more extreme than those of nonsynesthetes.

Our findings resemble those of previous studies of vowel–color mappings in smaller samples of (non)synesthetes, with lighter colors (yellow, green) being associated with more front vowels (higher F2; e.g., /i/, /e/), darker colors (e.g., red, brown, blue) being associated with back vowels (/o/, /u/), and redder colors being associated with low front vowels (e.g., /a/) (Marks, 1975; Wrembel, 2009; see Guillamon, 2014, for a cross-linguistic overview, particularly their Table 5, p. 44). The association of front vowels with high F2 to lighter colors could be related to more widespread cross-modal phenomena such as pitch–lightness associations (e.g., Ward et al., 2006), although this potential relationship requires further study, since vowel quality and pitch are independent (see Ohala, 1994, for an account that integrates these). Size–sound symbolism may also play a role: The space in the oral cavity is smaller for higher and more front vowels, which may in turn be associated with lightness and brightness (Cuskley & Kirby, 2013).

Second, color associations are predicted better by phonemes than by acoustic features, showing that cross-modal associations between vowel sounds and colors are modulated to an important degree by categorical perception (Goldstone & Hendrickson, 2010). Prior work on sound–color associations has mostly focused on how gradual changes in chromaticity are associated with low-level acoustic factors (Moos et al., 2014; Ward et al., 2006). Here we have shown that shifts in color associations correspond to category boundaries in participants’ vowel systems. The importance of categorical structure in vowel–color associations has implications for the underlying nature of synesthetic associations, pointing to the important role of learning (Mroczko-Wąsowicz & Nikolić, 2014; Simner, 2007).

For example, recent studies have shown that learning can play an influential role in synesthesia: Specifically, the presence of colored fridge magnets in childhood was formative for grapheme–color synesthesia (Witthoft & Winawer, 2013; Witthoft, Winawer, & Eagleman, 2015). However, the learning implied in the categorical effects that we observed is perhaps qualitatively different: Rather than being based on highly specific early perceptual experiences, our effects arise from a categorical warping of the vowel space that is a crucial part of spoken language acquisition.

Although our results indicate that acquired categories play a key role in vowel–color associations, there remains an important role for lower-level acoustic perceptual cues. For example, in the *u* dimension, we observed synesthetic effects for acoustic factors but not for phoneme category. This may point to the relevance of lower-level acoustic properties for some synesthetes specifically. It is possible that synesthetes react to acoustic factors in a way that nonsynesthetes do not, making acoustic factors a stronger predictor of color choices for synesthetes than for nonsynesthetes. Further targeted studies will be required in order to address this question.

Third, we have introduced a novel measure of structure in cross-modal associations. Most participants showed a significant degree of structure, implying that vowel–color associations rely at least in part on establishing structural isomorphism across perceptual domains. We found that this measure correlates with consistency across trials: Participants with more temporally consistent associations tend to have more structured mappings, with synesthetes being at the most consistent and structured extreme. Although synesthetic associations are idiosyncratic to some degree, the prevalence of structure shows that these associations share similarities with cross-modal associations in the general population (Simner et al., 2005). This implies that private, involuntary synesthetic associations, as well as overt, elicited cross-modal associations, may be underpinned to a significant degree by common principles of isomorphic mapping across sensory modalities. Our structure measure makes it possible to probe cross-modal and synesthetic associations in a way that is at least partly independent of consistency. This has the potential to provide a valuable complementary measure of genuineness in synesthesia (Simner, 2012). The structure measure can be applied in any domain in which the perceptual features of stimuli and responses (or of inducers and concurrents) are quantifiable in terms of some distance measure.

Fourth, we have shown that an online task can be used to learn about cross-modal associations and to infer synesthesia for a subset of participants along the way, providing a scalable method for identifying synesthetes and for studying the relation between cross-modal associations and synesthetic mappings from a population perspective. Although online tasks have become a fixture of synesthesia studies at least since Eagleman et al. (2007), they have mostly been used to test preselected sets of participants. Here we have shown that a widely advertised survey can succeed in capturing a broad and diverse sample of the population, including synesthetes. To foster more of this work, the code underlying our web application is openly available.

### Limitations and future work

Although acoustic and vowel phoneme predictors accounted for significant amounts of variation in color choices, as shown by the analyses above, the conditional *R*^2^ values in Table 2 indicate that individual variation accounted for at least as much variation in responses (the marginal-conditional *R*^2^ quantifies, roughly, the variance accounted for by both fixed and random effects). This indicates that, although there were obvious trends and although our structure measures point to a shared capacity for structure mappings, the specific identity of mappings may still vary considerably across participants. However, our structure results show that the capacity to structure mappings is strong and is shared across much of the population.

Further targeted studies should be done to tease apart the relative contributions of vowel category and grapheme category, especially the extent to which these may interact with how stimuli are presented. The data analyzed here were responses to auditory stimuli. Although there is evidence that auditory stimuli automatically activate graphemic representations to some extent (Ziegler & Ferrand, 1998), auditory presentation privileges acoustic features over graphemes (Cuskley et al., 2017), and graphemic representations may be less likely to be activated when auditory stimuli are presented devoid of a word-like context (e.g., within a nonword). Synesthetes tend to associate similar colors to similarly shaped graphemes (Brang, Rouw, Ramachandran, & Coulson, 2011; Eagleman, 2010; Jürgens, Mausfeld, & Nikolic, 2010; Watson, Akins, & Enns, 2012), implying an important role for visual grapheme shape in mediating the associations. However, other factors, such as ordinality in the alphabet and frequency in language, also play a role (e.g., van Leeuwen, Dingemanse, Todil, Agameya, & Majid, 2016; Watson et al., 2012).

Although most of the broad patterns found in Moos et al. (2014) were replicated in our data, the differences between the associations of synesthetes and nonsynesthetes was less pronounced than in their study (Fig. 5, and cf. Fig. 2 in Moos et al., 2014, p. 136). Two important differences between the studies are sheer sample size and online presentation, both of which added more variability to the responses in our study. Another possibility is that the confined set of 16 color swatches used by Moos et al. constrained responses in such a way that the divisions between synesthetes and nonsynesthetes became clearer. It may also be that Dutch speakers differ from English speakers in the finer details of how they map vowels onto color space. However, despite the differences, our results show quantitative differences between synesthetes and nonsynesthetes, as well as an important degree of shared mappings.

Although we expect the general role of categorical perception to be replicated across languages (mediated by known acoustic factors), we do not expect the precise groupings of vowel sounds to be replicated, because this is a function of how acoustic space is carved into a language-specific phoneme inventory. This opens up the possibility of a degree of linguistic relativity in cross-modal associations and synesthetic experience. Just as lexicalization patterns in the domain of color can shape low-level processes of color perception (Roberson, Pak, & Hanley, 2008), so phonemic structure may shape cross-modal associations. This is one place where linguistic diversity in phonetics, phonology, and orthography can be used to learn more about the mechanisms underlying vowel–color associations and to tease apart the roles of acoustic, phonemic, and graphemic features in cross-modal associations (Root et al., 2018; van Leeuwen et al., 2016).

Finally, further work will be needed to combine consistency scores with structure scores to create reliable behavioral indicators of genuine synesthesia, especially for use with large-scale online methodologies. Earlier reports of consistency scores had used them primarily to confirm self-identified synesthetes, rather than to detect synesthesia in a random sample. Figure 10a shows that our consistency cutoff to identify synesthetes (taken from Rothen et al., 2013) likely resulted in some false positives. A few potential issues may have contributed to this problem. First, Rothen et al. measured consistency in a grapheme–color task, which had 36 items. It may be that with fewer items (16 vowels), the consistency threshold was easier to pass. More importantly, consistency across items also needs to be taken into account: If a participant is consistent across trials and items, this is a likely flag for a false positive synesthete. Our structure measure captured this well, since cross-item distances form the core of the measure. Further work with confirmed synesthetes and nonsynesthetes will be needed in order to fully understand how best to combine structure and consistency scores to reliably detect synesthetes.

### Conclusions

In the first half of the 20th century, a common term for synesthesia was *audition colorée*, or “colored hearing”, after one of the most commonly reported forms of synesthesia: a connection between vowels and colors. Colored hearing was famously described by Nabokov (1989) and studied by such noted linguists as Roman Jakobson (Reichard, Jakobson, & Werth, 1949). This early focus on sound–color associations probably was one of the reasons for a fruitful period of experimental work on cross-modal correspondences between sound and color (e.g., Marks, 1978). In contrast, most modern work on synesthesia has tested graphemes in alphabetic writing systems (written representations of speech sounds), and indeed, grapheme–color synesthesia is likely by far the most studied variant of synesthesia to date.

Here we have brought these traditions together in pursuit of fundamental questions into the nature of synesthesia and cross-modal correspondences. We studied associations between vowel sounds and colors in over 1,100 people, including hundreds of synesthetes. We replicated earlier findings on the relation between acoustic cues and color choices, but additionally showed an important role for categorical perception over and above such cues. Our findings underline the roles of learned categories and structural isomorphisms in the cross-modal associations made by synesthetes and nonsynesthetes alike. The measure of structural isomorphism we have introduced can help create more nuanced diagnostic tools for synesthesia. As work on synesthesia and cross-modal associations grows to accommodate larger samples and more varied measures, it will provide fundamental insights into how mappings across sensory modalities are made and maintained.

## Appendix 1: Audio file categorization

**Table 6:** F1 and F2 values of audio stimuli, as well as canonical Dutch phoneme categories and graphemes. F1 and F2 values of assigned Dutch phonemes are also listed

Stimulus number	Original Moos et al. (2014)		Canonical Dutch by phoneme Category from Adank et al. (2007)		Phoneme category	Grapheme category
	F1	F2	F1	F2		
1	231	2,324	291.75	2,378	i	i
2	279	2,328	291.75	2,378	i	i
3	356	2,365	291.75	2,378	i	i
4	479	2,179	415.50	2,187.75	e	e
5	628	2,038	394.75	2,013.75		i
6	706	1,833	516.5	1,819.25	ε	e
7	767	1,561	791.75	1,516.5	a	a
8	695	1,176	654	1,195	ɑ	a
9	580	834	423.5	894		o
10	491	759	423.5	894		o
11	323	632	423.5	894		o
12	371	649	423.5	894		o
13	361	545	423.5	894		o
14	283	445	423.5	894		o
15	222	774	283	935	u	œ
16	228	1,524	408.25	1,654	ø	u

## Appendix 2: Grapheme predictor models

### Lightness (*L*)

Formula: *L* ~ graphcat × graphSyn + (1 | Participant) + (1 | Trial)

**Table:**

Random effects:			
Groups	Name	Variance	Std. Dev.
Participant	(Intercept)	72.8006 8.5323	
Trial	(Intercept)	0.1121	0.3348
Residual		266.0102 6.3098	
Number of obs: 52,320	groups: profileid	1,130; Trial	3
Fixed effects:			
	Estimate	*SE*	*t* Value
(Intercept)	58.43613	1.30452	44.795
graphcat[e]	6.58091	1.13204	5.813
graphcat[i]	10.37372	0.97758	10.612
graphcat[o]	– 9.50099	0.92226	– 10.302
graphcat[u]	8.18896	1.39438	5.873
graphcat[œ]	– 11.44930	1.38716	– 8.254
graphSyn[No]	– 0.08566	1.54476	– 0.055
graphSyn[Unknown]	0.74399	1.34078	0.555
graphcat[e] × graphSyn[No]	– 2.16767	1.35470	– 1.600
graphcat[i] × graphSyn[No]	– 2.62095	1.1698	– 2.240
graphcat[o] × graphSyn[No]	3.0334	1.10324	2.750
graphcat[u] × graphSyn[No]	– 2.29062	1.66649	– 1.375
graphcat[œ] × graphSyn[No]	2.05050	1.65938	1.236
graphcat[e] × graphSyn[Unknown]	– 1.52903	1.17683	– 1.299
graphcat[i] × graphSyn[Unknown]	– 1.49775	1.01636	– 1.474
graphcat[o] × graphSyn[Unknown]	0.19959	0.95891	0.208
graphcat[u] × graphSyn[Unknown]	– 2.22422	1.44914	– 1.535
graphcat[œ] × graphSyn[Unknown]	0.02415	1.44240	0.017

### Green–red (*u*)

Formula: *u* ~ graphcat × graphSyn + (1 | Participant) + (1 | Trial)

**Table:**

Random effects:			
Groups	Name	Variance	Std. Dev.
profileid	(Intercept)	3.435e+02	18.53454
Trial	(Intercept)	2.321e–03	0.04817
Residual		2.619e+03	51.17159
Number of obs: 52,320	groups:	profileid,1130	Trial,3
Fixed effects:			
	Estimate	*SE*	*t* Value
(Intercept)	31.1034	3.3360	9.324
graphcat[e]	– 30.4597	3.5516	– 8.576
graphcat[i]	– 27.0697	3.0670	– 8.826
graphcat[o]	– 7.0261	2.8935	– 2.428
graphcat[u]	– 21.2296	4.3746	– 4.853
graphcat[œ]	1.1076	4.3520	0.254
graphSyn[No]	– 9.7412	3.9929	– 2.440
graphSyn[Unknown]	– 5.9603	3.4673	– 1.719
graphcat[e] × graphSyn[No]	10.8361	4.2502	2.550
graphcat[i] × graphSyn[No]	8.0058	3.6703	2.181
graphcat[o] × graphSyn[No]	6.2345	3.4612	1.801
graphcat[u] × graphSyn[No]	7.9684	5.2283	1.524
graphcat[œ] × graphSyn[No]	– 2.9329	5.2061	– 0.563
graphcat[e] × graphSy[Unknown]	9.1329	3.6921	2.474
graphcat[i] × graphSyn[Unknown]	5.2102	3.1886	1.634
graphcat[o] × graphSyn[Unknown]	– 0.8595	3.0084	– 0.286
graphcat[u] × graphSyn[Unknown]	1.9343	4.5463	0.425
graphcat[œ] × graphSyn[Unknown]	– 11.9767	4.5253	– 2.647

### Blue-yellow (*v*)

Formula: *v* ~ graphcat × graphSyn + (1 | Participant) + (1 | Trial)

**Table:**

Random effects:			
Groups	Name	Variance	Std. Dev.
profileid	(Intercept)	608.9975	24.6779
Trial	(Intercept)	0.2484	0.4984
Residual		3408.6133	58.3833
Number of obs: 52,320	groups:	profileid,1130	Trial,3
Fixed effects:			
	Estimate	*SE*	*t* Value
(Intercept)	6.0206	4.1046	1.467
graphcat[e]	14.2310	4.0522	3.512
graphcat[i]	23.1225	3.4993	6.608
graphcat[o]	– 16.5514	3.3013	– 5.014
graphcat[u]	18.0172	4.9912	3.610
graphcat[œ]	– 10.1347	4.9654	– 2.041
graphSyn[No]	1.2632	4.9018	0.258
graphSyn[Unknown]	2.3055	4.2557	0.542
graphcat[e] × graphSyn[No]	– 5.9137	4.8493	– 1.220
graphcat[i] × graphSyn[No]	– 2.9596	4.1876	– 0.707
graphcat[o] × graphSyn[No]	– 4.6218	3.9491	– 1.170
graphcat[u] × graphSyn[No]	– 0.8866	5.9653	– 0.149
graphcat[œ] × graphSyn[No]	– 13.5783	5.9399	– 2.286
graphcat[e] × graphSyn[Unknown]	– 2.7891	4.2125	– 0.662
graphcat[i] × graphSyn[Unknown]	– 2.8695	3.6381	– 0.789
graphcat[o] × graphSyn[Unknown]	– 7.1203	3.4325	– 2.074
graphcat[u] × graphSyn[Unknown]	– 4.1091	5.1872	– 0.792
graphcat[œ] × graphSyn[Unknown]	– 12.6501	5.1631	– 2.450

## Appendix 3: Pairwise contrasts for vowel category model

### Vowel Contrasts

All results are averaged over the levels of: SynVowel. Confidence level used: .95; Conf-level adjustment: Bonferroni method for 36 estimates; *p* value adjustment: Bonferroni method for 36 tests.

### Synesthete–Nonsynesthete contrasts

Synesthete–Nonsynesthete contrasts (*Estimate* indicates difference with Synesthetes as the intercept). Degrees-of-freedom method: Satterthwaite; Confidence level used: .95; Conf-level adjustment: Bonferroni method for two estimates; *p* value adjustment: Bonferroni method for two tests.

### Lightness (*L*)

**Table:**

Phoneme	Estimate	*SE*	*df*	Lower CL	Upper CL	*t* Ratio	*p* Value
a	– 1.3425405	0.8207041	4890.22	– 2.9514891	0.2664081	– 1.636	.1019
ɑ	1.4107140	0.8158002	4778.76	– 0.1886209	3.0100488	1.729	.0838
e	– 2.5960336	0.8180872	4831.38	– 4.1998520	– 0.9922152	– 3.173	.0015
ε	3.4268784	0.8204578	4881.72	– 5.0353442	– 1.8184126	– 4.177	< .0001
i	– 4.3373060	0.6468907	1937.04	– 5.6055023	– 3.0691097	– 6.705	< .0001
	– 2.7163752	0.8162240	4789.28	– 4.3165410	– 1.1162095	– 3.328	.0009
ø	– 5.9132909	0.8182063	4845.37	– 7.5173428	– 4.3092389	– 7.227	< .0001
	1.6846702	0.5983915	1419.51	0.5115540	2.8577863	2.815	.0049
u	0.3076004	0.8230901	4942.52	– 1.3060260	1.9212268	0.374	.7086

### Green-Red (*u*)

There was no significant main effect of synesthetic status in the *u* dimension, so comparisons between synesthetes and nonsynesthetes were not calculated.

### Blue-Yellow (*v*)

**Table:**

Phoneme	Estimate	*SE*	*df*	Lower CL	Upper CL	*t* Ratio	*p* Value
a	– 6.922706	2.709932	7418.02	– 12.2349421	– 1.61047039	– 2.555	.0107
ɑ	– 5.299904	2.690772	7226.73	– 10.5745819	– 0.02522639	– 1.970	.0489
e	– 7.304734	2.699739	7317.78	– 12.5969884	– 2.01247963	– 2.706	.0068
ε	– 11.170913	2.708874	7400.58	– 16.4810748	– 5.86075175	– 4.124	< .0001
i	– 9.354233	2.015447	2398.50	– 13.3050817	– 5.40338345	– 4.641	< .0001
	– 6.094542	2.692466	7246.25	– 11.3725388	– 0.81654528	– 2.264	.0236
ø	– 13.694378	2.700581	7351.95	– 18.9882834	– 8.40047350	– 5.071	< .0001
	2.790157	1.812159	1571.02	– 0.7621881	6.34250210	1.540	.1238
u	2.302293	2.719161	7504.97	– 3.0280339	7.63261997	0.847	.3972

## Footnotes

1 Note the slightly confusing terminology here: High vowels have a lower value of F1, whereas low vowels have a higher value of F1. This is because the high/low distinction as applied to vowel quality refers to the position of the tongue in articulation (rather than to the F1 value): A high tongue makes for a lower value of F1, whereas a low tongue makes for a higher value of F1. For a detailed overview of the articulatory acoustics of vowel production, see Johnson (2011).

2 The survey also included the question “Do you have synesthesia?,” which was answered by 875 participants, with 73 saying they did have synesthesia, 290 saying they did not, and 512 saying they didn’t know. Since this question did not specify what particular type of synesthesia a participant had, these responses were not used in the present analysis. This questionnaire did not screen for color blindness, but this is unlikely to have drastically affected our participant pool, since color blindness is X-linked recessive, and our participants were predominantly female. On the basis of the reported gender for a large subset of the sample and rates of color blindness (8% in males and 0.05% in females; Chan, Goh, & Tan, 2014), our sample likely had approximately 25–30 colorblind participants, which is a small percentage of our dataset (~ 2%), and one that was likely to add noise to the results rather than to inadvertently support our hypotheses.

3 In the original Dutch: *Vowel–color task:* “In deze test koppel je kleuren aan taalklanken. Je hoort een geluid, en dan kies je de kleur die je daar het beste bij vindt passen. Synestheten zien altijd dezelfde kleur voor dezelfde klank: is dat bij jou ook zo? Probeer zo intuïtief mogelijk te reageren.” *Grapheme–color task:* “In deze test koppel je wederom kleuren aan letters, maar dan iets preciezer. Je ziet een letter of cijfer, en dan kies je de kleur die je daar het beste bij vindt passen. Probeer zo intuïtief mogelijk te reageren.”

4 ∆*E*_2000_ distances may also be more accurate for detecting synesthesia using consistency scores. However, we were unable to use this measure to detect synesthetes, because there is no benchmark for this in the literature. Rothen et al. (2013) provided an evaluation of CIELab-based consistency scores that used straight Euclidean distance in CIELab space (also known as ∆*E*_1976_), but they did not test ∆*E*_2000_. They reported superior performance in detecting synesthetes using CIELuv distances, so these were preferred for our consistency scores and were used to classify synesthetes in the sample. However, for detecting structural isomorphism, we used ∆*E*_2000_ because this is likely to better capture real perceptual distances.

5 Participants’ *z* scores and *p* values are provided as part of their structure score in the data repository at https://github.com/mdingemanse/colouredvowels, along with Python code to calculate pairwise distances and run the Mantel simulations. However, note that if recomputed, these values would differ very slightly for each participant, since they are based on a simulated distribution created when the scores were calculated. Recomputing these values would also involve creating new distributions, which would slightly alter *exactly* where participants fall on those distributions. However, the overall picture described in this section would hold.
